# Intrapulmonary metastasis from primary pulmonary meningioma presenting as multiple cystic lesions: a case report

**DOI:** 10.1186/s12890-018-0773-7

**Published:** 2019-01-08

**Authors:** Xin Wang, Pengfei Li, Ping Zhou, Yiyun Fu, Yutian Lai, Guowei Che

**Affiliations:** 1 Department of Thoracic Surgery, West China Hospital, Sichuan University, Chengdu, 610041 People’s Republic of China; 2 Department of Pathology, West China Hospital, Sichuan University, Chengdu, 610041 People’s Republic of China

**Keywords:** Pulmonary meningioma, Computed tomography, Cystic, Metastasis

## Abstract

**Background:**

Cystic lung lesions involving both lungs include a variety of diseases, such as pulmonary Langerhans cell histiocytosis, lymphangioleiomyomatosis and pulmonary metastasis. Primary pulmonary meningioma accompanied with intrapulmonary metastasis was extremely rare and we were not aware of previous studies reporting with cystic radiological manifestation.

**Case presentation:**

A 64-year-old female patient was admitted to our department for a mass located in right posterior mediastinum with multiple cystic pulmonary lesions. A thoracoscopic lung biopsy was performed and the diagnosis was confirmed as bilateral pulmonary metastasis from primary pulmonary meningioma.

**Conclusions:**

Intrapulmonary metastasis from a primary pulmonary meningioma may manifest as multiple thin-walled cystic lesions on computed tomography. Differential diagnosis of cystic pulmonary disease should include this situation. Our case shows the new CT manifestation of metastatic primary pulmonary meningioma and the importance of immunomolecular analysis.

## Background

Cystic lung lesions involving both lungs include a variety of diseases, such as pulmonary Langerhans cell histiocytosis, lymphangioleiomyomatosis, Birt Hogg Dubé syndrome and pulmonary metastasis [1]. Meningioma might occur in extracranial organs and pulmonary meningioma is the most reported since it introduced by Weiss et al. in 1952 [2]. The primary pulmonary meningioma (PPM) with metastasis was extremely rare, which usually presented as isolated nodules rather than bilateral lesions. We describe a patient with one major lesion and multiple cystic lesions scattered over both lungs. The major mass was diagnosed as primary pulmonary meningioma and other cystic lesions was classified as intrapulmonary metastasis after thoracoscopic surgery.

## Case report

A 64-year-old female patient was admitted to our department for multiple pulmonary lesions discovered by health examination for 1 month. She was asymptomatic, in good health, and had no history of pulmonary or neurologic disease. She denied history of tuberculosis, and she was a non-smoker.

**Fig. 1:**
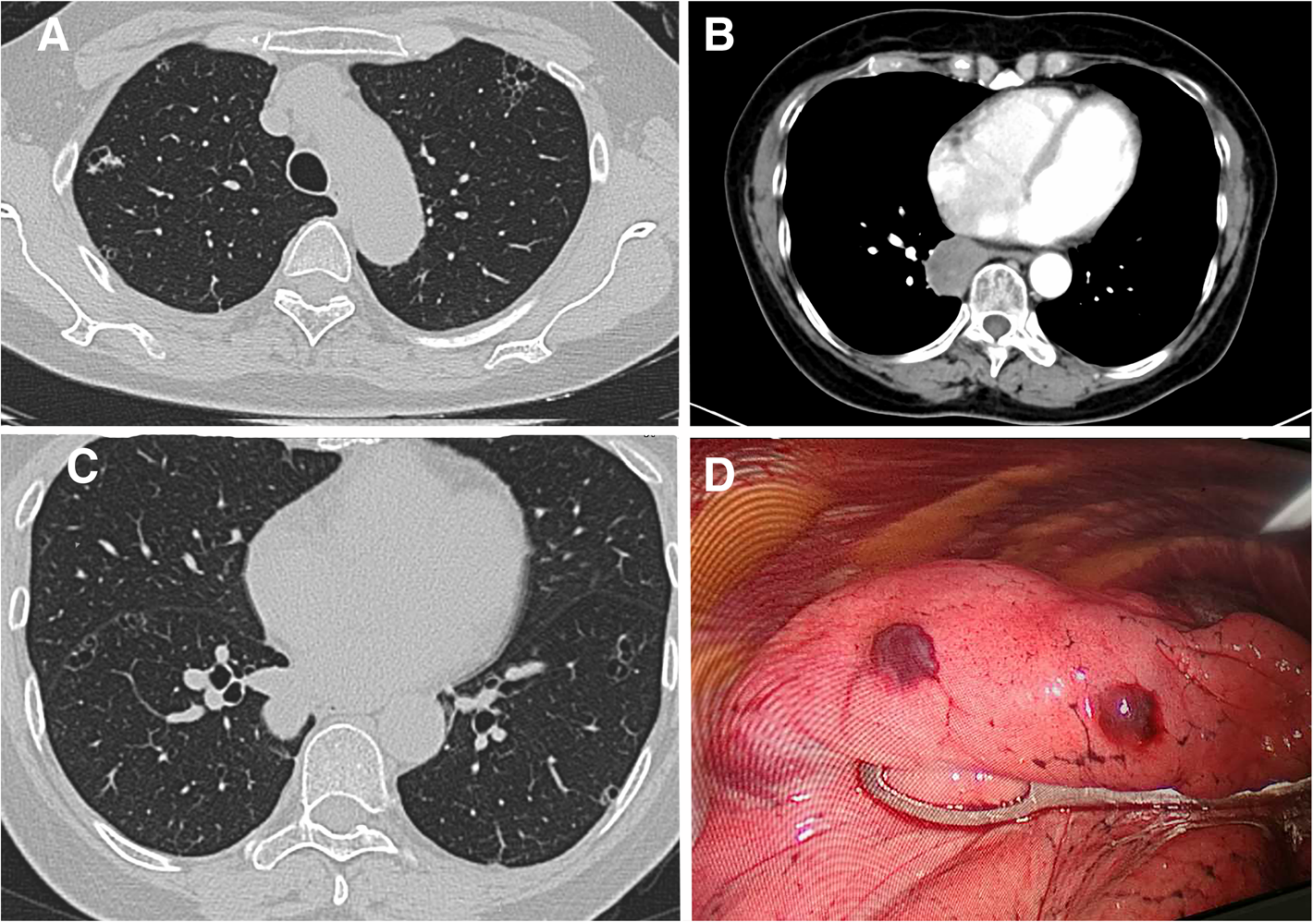
**a**, **c**: CT revealed multiple cycstic lesions involving both lungs. **b**: Major mass located in the right posterior mediastinum. **d**: Lung biopsy was taken for pathological examination

Chest computed tomography (CT) revealed multiple thin-, smooth-walled cysts or cystic nodules with solid component were scarred within the lung parenchyma, sized from 0.8 cm to 2 cm (Fig. 1a, c). Enhanced CT scan revealed a 3.4 cm (cm) rounded mass located in the right posterior mediastinum at the inferior pulmonary vein plane (Fig. 1b). It showed slight enhancement in the enhanced CT scan. The result of preoperative brain magnetic resonance imaging was also negative.

Thoracoscopic lung resection was scheduled for this patient. During the operation, the larger mass, which enveloped by fat-like thin films, was located on the surface of right lower lobe rather than the mediastinum. The tumor was stripped out successfully with a right-angle electrode and we also wedge resected two cystic lesions for pathological examination (Fig. 1d).

**Fig. 2:**
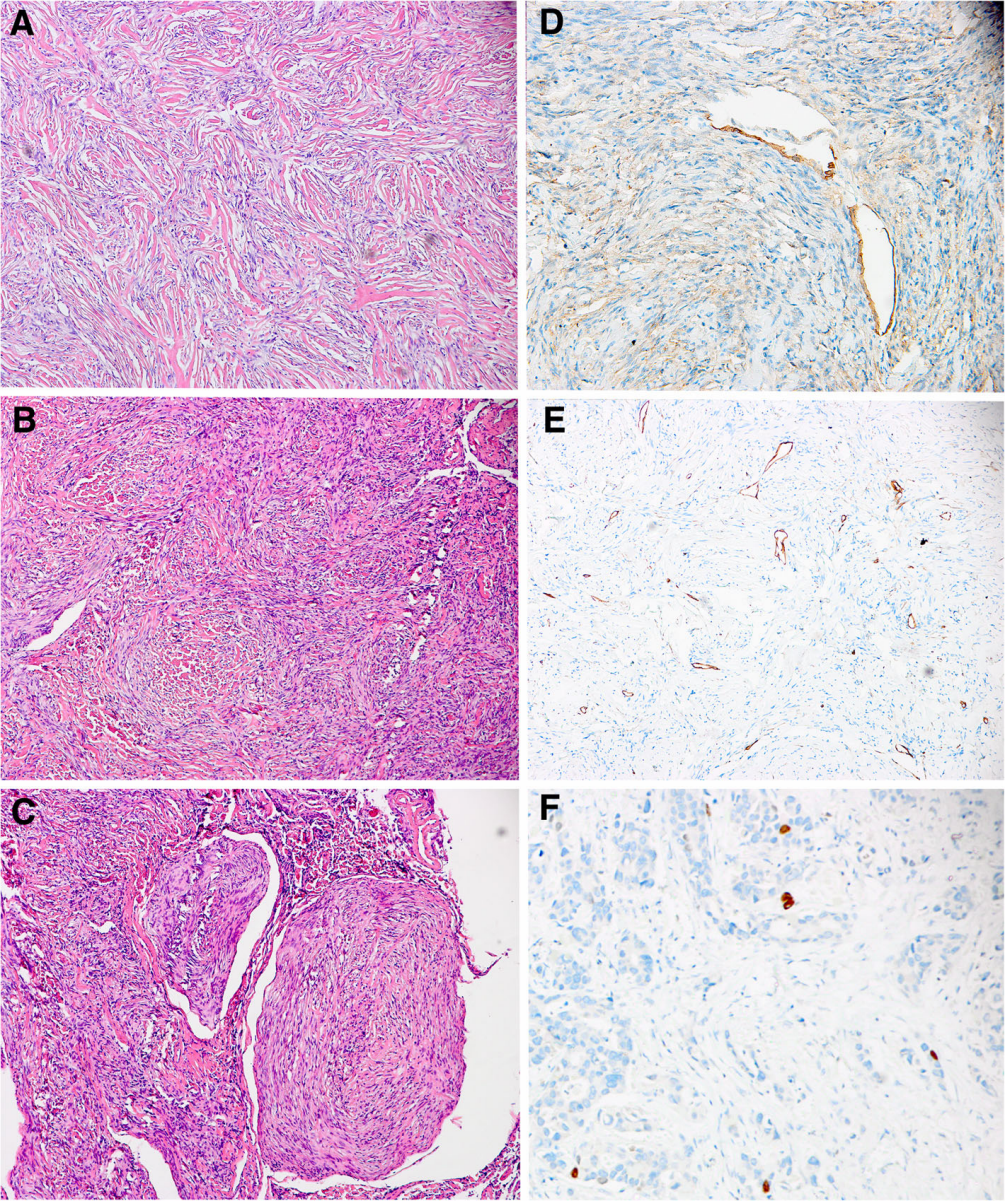
**a**, **b**, **c**: Tumor consisted of spindle cells arranged in swirls, Hematoxylin & eosin, × 100. **d**: IHC staining of EMA was positive, X200. **e**: IHC staining of CD34 was positive, X100. **f**: IHC staining of Ki-67/MIB-1 was positive, X200

Routine pathological examination of both major mass and cystic lesions revealed the tumor consisting of spindle cells arranged in swirls scattered with a small amount of typical psammoma body (Fig. 2a, b, c hematoxylin and eosin staining, X100). Immunohistochemical (IHC) staining was performed for both major mass and cystic lesions and all lesions were positive for epithelial membrane antigen (Fig. 2d, X200), CD34(Fig. 2e, X100), progesterone receptor (PR), Ki-67 and negative for STAT6 and CD68. The Ki-67/MIB-1 labeling index was less than 2% (Fig. 2f, X200).

The patient underwent an uneventful postoperative course and no additional therapy was added for her.

## Conclusion

Intrapulmonary metastasis from a primary pulmonary meningioma may manifest as multiple thin-walled cystic lesions on computed tomography. Differential diagnosis of cystic pulmonary disease should include this situation. Our case shows the new CT manifestation of metastatic primary pulmonary meningioma and the importance of immunomolecular analysis.

## Discussion

The diagnosis of the PPM depends on pathological and radiological studies, and more importantly, excluding metastasis from central nervous system. It is frequently reported that intracranial meningioma could metastases to the lungs, but only one case reporting multiple primary pulmonary meningioma [3]. The radiology manifestation of almost all of the metastasis or primary pulmonary meningioma showed as well-circumscribed solid nodules or mass [4, 5]. We are not aware of previous reports of intrapulmonary metastasis from primary pulmonary meningioma presenting as multiple thin-walled cystic lesions scattered within the lung parenchyma.

The initial diagnosis of this patient was considered as mediastinal tumor with multiple thin-walled cysts involving bilateral lungs based on chest CT. These thin-walled cysts were suspected to be lymphangioleiomyomatosis or Langerhans cell histiocytosis for their radiology manifestations. Morphological and histological characters ruled out the possibility of lymphangioleiomyomatosis. And negative expression of IHC staining of CD68 ruled out the diagnosis of Langerhans cell histiocytosis. Other IHC markers, such as KI67/MIB-1, CD34 and PR evaluated at the same time because these markers are correlated with recurrence and metastasis^4^. The results of pathology examination suggested to be benign primary pulmonary meningioma, however, even benign meningioma could metastasize [6]. And of these rare metastatic benign diseases, Stefani et al. reported that an intracranial meningioma could metastasize to bilateral lungs presenting as defuse cystic lesions [7]. Given the above consideration, the scattered cystic lesions in our patient were classified as multiple intrapulmonary metastasis from primary pulmonary meningioma which has not previously been reported.

## Abbreviations

CD: Clusters of Differentiation
cm: Centimeter
CT: computed tomography
EMA: Epithelial membrane antigen
IHC: Immunohistochemical
MRI: Magnetic Resonance Imaging
PPM: primary pulmonary meningioma
PR: Progesterone receptor

## References

[1] Raoof S, Bondalapati P, Vydyula R (2016). Cystic lung diseases: algorithmic approach. Chest.

[2] Weiss A, Philippides D, Montrieul B (1952). Primary pulmonary meningioma: a rare, slow-growing tumor. J Radiol Electrol Arch Electr Medicale.

[3] Satoh Y, Ishikawa Y (2010). Primary pulmonary meningioma: ten-year follow-up findings for a multiple case, implying a benign biological nature. J Thorac Cardiovasc Surg.

[4] Incarbone M, Ceresoli GL, Di Tommaso L (2008). Primary pulmonary meningioma: report of a case and review of the literature. Lung cancer (Amsterdam, Netherlands).

[5] Surov A, Gottschling S, Bolz J (2013). Distant metastases in meningioma: an underestimated problem. J Neuro-Oncol.

[6] Bohra H, Rathi KR, Dudani S, Bohra A, Vishwakarma S, Sahai K. The study of mib-1 li and cd 34 as a marker of proliferative activity and angiogenesis in different grades of meningioma. J Clin Diagn Res 2016;10(8):Ec14–17. 10.7860/JCDR/2016/12690.8328 PMC5028582 27656445

[7] Stefani A, Rossi G, Pecchi A (2013). An unusual case of cystic interstitial lung disease. Lancet.

